# Texture dependence of motion sensing and free flight behavior in blowflies

**DOI:** 10.3389/fnbeh.2012.00092

**Published:** 2013-01-11

**Authors:** Jens P. Lindemann, Martin Egelhaaf

**Affiliations:** Neurobiology and CITEC, Bielefeld UniversityBielefeld, Germany

**Keywords:** vision, motion detection, texture, free flight, behavior, model, simulations, insects

## Abstract

Many flying insects exhibit an active flight and gaze strategy: purely translational flight segments alternate with quick turns called saccades. To generate such a saccadic flight pattern, the animals decide the timing, direction, and amplitude of the next saccade during the previous translatory intersaccadic interval. The information underlying these decisions is assumed to be extracted from the retinal image displacements (optic flow), which scale with the distance to objects during the intersaccadic flight phases. In an earlier study we proposed a saccade-generation mechanism based on the responses of large-field motion-sensitive neurons. In closed-loop simulations we achieved collision avoidance behavior in a limited set of environments but observed collisions in others. Here we show by open-loop simulations that the cause of this observation is the known texture-dependence of elementary motion detection in flies, reflected also in the responses of large-field neurons as used in our model. We verified by electrophysiological experiments that this result is not an artifact of the sensory model. Already subtle changes in the texture may lead to qualitative differences in the responses of both our model cells and their biological counterparts in the fly's brain. Nonetheless, free flight behavior of blowflies is only moderately affected by such texture changes. This divergent texture dependence of motion-sensitive neurons and behavioral performance suggests either mechanisms that compensate for the texture dependence of the visual motion pathway at the level of the circuits generating the saccadic turn decisions or the involvement of a hypothetical parallel pathway in saccadic control that provides the information for collision avoidance independent of the textural properties of the environment.

## Introduction

For flying insects the visual system is the primary source of information about their environment (for review see Egelhaaf, 2006; Egelhaaf et al., 2012). In ego-motion estimation and flight stabilization visual information is complemented by mechano-sensory input (Hengstenberg, 1993), but airborne insects rely exclusively on vision for tasks like altitude control, estimation of flight distance over ground or obstacle avoidance. They most likely extract the relevant information from the characteristic movements of the retinal image, the optic flow, induced during locomotion (Egelhaaf, 2006). During translational locomotion in a static environment the speeds of the perceived retinal movements are proportional to the translation velocity of the animal and inversely proportional to the distance to objects in the environment. In contrast, rotational movements generate retinal velocities independent of the depth structure of the environment (Koenderink and van Doorn, 1987). Flies, but also other insects and even those birds that have been analyzed in this regard, show a flight strategy which apparently separates these two components already on a behavioral level. The flight is actively structured in sequences of short and fast shifts of gaze direction (saccades) alternating with flight phases during which the gaze is stabilized against rotations (Schilstra and van Hateren, 1998, 1999; van Hateren and Schilstra, 1999; Eckmeier et al., 2008; Boeddeker et al., 2010; Braun et al., 2010, 2012; Geurten et al., 2010).

Optic flow processing of flying insects, and especially of flies, has been described in great detail (for review see e.g., Egelhaaf, 2006; Borst et al., 2010). Retinal image motion is detected by local motion detection circuits which project on spatially integrating tangential cells in the 3rd visual neuropile, the lobula plate. When stimulated with the optic flow that an animal has seen during free flight, these lobula plate tangential cells (LPTCs) respond to the translational optic flow between saccadic turns in a distance dependent manner (Kern et al., 2005; Karmeier et al., 2006; Liang et al., 2012). Therefore, LPTCs may play a role in providing the animal with spatial information relevant, for instance, in the context of collision avoidance. However, extracting spatial information from translational optic flow is complicated by the non-linear and ambiguous encoding of motion information by LPTCs (e.g., Egelhaaf et al., 1989; Kern et al., 2005; Wu et al., 2012). For instance, their responses are strongly pattern dependent (Egelhaaf and Borst, 1989; Egelhaaf et al., 1989). Although these pattern dependencies are, on average, much reduced under stimulation with natural images with their typical broad-band spatial frequency spectra (Dror et al., 2001; Straw et al., 2008), the time course of individual response traces shows marked pattern dependent modulations (Egelhaaf et al., 1989; Meyer et al., 2011; O'Carroll et al., 2011). Hence responses of LPTCs contain components which are unrelated to the optic flow, but reflect the textural properties of the environment.

The response properties of LPTCs and of visual motion processing presynaptic to them can be reproduced by simulation models at a quantitative level (Lindemann et al., 2005; Hennig et al., 2011; Hennig and Egelhaaf, 2012). All these models are based on the elementary motion detection algorithm as proposed by Hassenstein and Reichardt (1956; Reichardt, 1961). Being computationally lightweight compared to computer vision algorithms for the estimation of dense optic flow fields, bio-inspired motion detection schemes are interesting for robot vision. Accordingly, there have been various attempts to implement visual robot control based on this principle. Among these only few systems replicate the saccadic flight behavior in tethered robotics (Reiser and Dickinson, 2003) or closed-loop simulation (Dickson et al., 2008; Lindemann et al., 2008; Stewart et al., 2010).

A recent attempt to model collision avoidance behavior in a simulated flight arena (CyberFly) was based on a model of the fly visual motion pathway with just two LPTCs as output neurons, one in each half of the visual system, which respond best to front-to-back motion in large parts of the visual field (Lindemann et al., 2008). Based on the responses of the two simulated LPTCs, the CyberFly generates saccadic flight trajectories in a closed-loop simulation. The system was tuned to avoid the wall of a virtual flight arena covered with an appropriately blurred random checker board. However, it fails in a geometrically identical setup textured with a similar random checker board made from smaller squares. Hence, the performance of this model depends on the texture of the walls. This result may be explained by the contrast and spatial frequency dependence of fly motion detector responses, although the statistical properties of the tested wall patterns change only moderately by spatial scaling similar to natural images (van der Schaaf and van Hateren, 1996) and the responses of LPTCs to such patterns have been suggested to depend only little on their specific texture (Dror et al., 2001; Straw et al., 2008).

Here we show that the unexpectedly strong texture dependence of the closed-loop performance of the CyberFly can be attributed to a texture-dependent inversion of the direction of saccadic turns of the CyberFly at certain locations of the simulated arena that is caused by a qualitative change in the underlying simulated LPTC responses. We ensured that this feature is no artifact of the model by monitoring the responses of fly LPTCs to the same visual stimuli.

This pattern dependence of LPTCs and the current CyberFly is in apparent contrast to the behavioral performance of real flies. When confronted with similar environments as used in our model simulations and electrophysiological experiments (Kern et al., 2000, 2012; Tammero and Dickinson, 2002a), flies can be assumed to easily deal with all scales of random checker board textures without colliding with the walls of the flight arena. Nonetheless, the details of flight behavior may depend on the texture of the flight arena (e.g., Frye and Dickinson, 2007). Therefore we scrutinized free flight behavior of blowflies in a cylindrical arena covered with the same random patterns as used in our model simulations and electrophysiological experiments. Although the flies are able to cope well with both textures, subtle differences in the flight structure can be associated to the different textures. The divergent dependence of neuronal and behavioral performance on the textural properties of the environment will be interpreted on the basis of our CyberFly model as well as alternative possibilities. The strong texture dependence of insect motion detection and optic flow processing mechanisms, so far, prevent their successful application in robotics. Understanding how insects cope with this problem may be a key to optic flow based reactive control of mobile robots.

## Materials and methods

### Stimulus generation

Model simulations and electrophysiological recordings described below use retinal images generated during simulated flights in a cylindrical arena for stimulation. The cylinder was alternately covered by two checkerboard textures differing in size and number of the texels (black and white squares). The coarse pattern was composed of 36 × 11 squares corresponding to 10° edge length, if viewed from the center of the cylinder. The fine pattern contains 144 × 44 squares corresponding to 2.5° edge length. An intermediate texture having an edge length of 5° was used in simulation experiments only.

The simulated flight followed a trajectory composed of straight segments and sharp 45° turns mimicking the saccadic flight style of flies. The resulting octagonal trajectory follows the wall of the cylindrical arena at half height. Forward speed was set to 1 m/s for the electrophysiological recordings and varied between 0.5 and 1.25 m/s in simulation. The minimum distance of the trajectory from the arena center was 358 mm in the electrophysiological experiment. For simulation two additional trajectories were used with translational segments shortened by a factor of 2 or 4, resulting in distances of 207 and 135 mm, respectively. Turn velocities were the same for all trajectories and correspond to 45° saccadic turns; these saccade characteristics are well within the range observed by Schilstra and van Hateren (1999), van Hateren and Schilstra (1999).

Using computer graphics (ivRender, http://opensource.cit-ec.de/projects/ivtools), we rendered the retinal input for presentation to the model or the animal, respectively.

### Model simulations

For the model simulations we used the sensory part of the closed-loop CyberFly agent described earlier (Lindemann et al., 2005, 2008). The model has a rectangular matrix of input elements spaced horizontally and vertically by 2° visual angle. The simulated 3D-environment was sampled by the renderer at a rate of 1 kHz, applying Gaussian filter masks (σ = 2°) to the rendered image. A lateral inhibition of neighboring elements was applied as a second spatial filter (Lindemann et al., 2012). The resulting time-dependent signals of the simulated input elements were convolved with a temporal linear filter approximating the filter properties of cells in the 1st visual neuropile of the fly, the lamina monopolar cells (LMCs). The temporally filtered signals of horizontally neighboring inputs were then fed into a correlation-type motion detector built from first-order temporal high- and low-pass filters and a multiplication stage. The output of an array of these detectors was weighted according to the local sensitivity of the modeled LPTCs, the HSE cells (Krapp et al., 2001) and spatially integrated by an electrical equivalent circuit of a passive membrane patch to emulate the gain control properties of these neurons (Borst et al., 1995). The parameters of the system were tuned by quantitative comparison of the model responses with the responses to behaviorally generated optic flow of a particular blowfly lobula plate tangential cell, the HSE cell (Lindemann et al., 2005).

### Electrophysiological experiments

One- to three-days-old female blowflies (*Calliphora vicina*) were taken from stocks of the department. Dissection was done according to the procedure described in Dürr and Egelhaaf (1999). The experiments were carried out at room temperatures between 24 and 34°C, which correspond to the head temperature of blowflies during flight (Stavenga et al., 1993). Intracellular recordings from the axon of HS-cells, a specific class of LPTCs, in the right optic lobe (lobula plate) were done with sharp glass electrodes (G100TF-4, Warner Instruments Inc.) pulled on a Flaming/Brown micropipette puller (Sutter Instruments P-97). The resistance of the electrodes, filled with 1 M KCl, was 20–50 MΩ. Ringer solution (as specified in Kurtz et al., 2000) was used to prevent the neuronal tissue from desiccation. Recordings were sampled at 4 kHz (DT 3001, Data Translation, Marlboro, MA) and stored on hard disk for offline analysis.

Stimuli were generated as described above and presented in the FliMax stimulus device at an update rate of 368 Hz (Lindemann et al., 2003). An approximation of the response of the left HS-cells was obtained by presenting a mirrored version of the reconstructed image sequences to HS-cells in the right half of the visual system (Kern et al., 2005).

### Behavioral experiments

Behavioral experiments were performed in a cylindrical arena made from aluminum (diameter 70 cm, height 50 cm). The cylinder was papered with one of two different textures equivalent to those used in the simulation analysis. Textures were printed red on white to have strong contrast for the green sensitive motion vision pathway of the flies (Yamaguchi et al., 2008) while having only low contrast for the red sensitive cameras. The arena floor made from acrylic glass was covered with parchment paper. Black gauze covered the cylinder to keep flies inside while permitting filming from above. Two cameras (CR600×2, Optronis GmbH, Kehl, Germany) were mounted above the arena. One camera was centered above the arena and its optical axis was aligned with the symmetry axis of the arena wall. The second camera was tilted and shifted to generate a stereo-view of the setup. The cameras were synchronized and recorded the fly behavior at a rate of 500 frames per second. Light was provided by 3 LED panels (Marathon MultiLED, GS Vitec GmbH, Gelnhausen, Germany) illuminating the scene from above and 12 halogen bulbs mounted beneath the translucent arena floor. Lights were adjusted to give a largely homogenous illumination. The recorded flight sequences were stored on hard disc and analyzed offline using the ivTrace software package (http://opensource.cit-ec.de/projects/ivtools). This analysis resulted in 2D trajectories for each flight and camera view, respectively. Stereo-calibration of the camera arrangement and 3D-reconstruction of the fly position from two corresponding 2D trajectories was done using Matlab (The Mathworks Inc., Natick, MA, USA) and the Camera Calibration Toolbox for Matlab (http://www.vision.caltech.edu/bouguetj/calib_doc). Body yaw orientation was extracted from the top-view using ivTrace.

Female blowflies (*Lucilia sericata*) were released into the arena through a hole in the center of the arena floor. The recording was started when the flies were flying and stopped either when the fly had landed or when the camera recording buffer was full. To exclude immediate starting and landing phases from the analysis, trajectory sections preceding the first saccade and succeeding the last detected saccade (detection criteria given below) were omitted from the further analysis. Flies landing in the arena were flushed by waving hands above the arena. 113 flights of six different animals were analyzed (56 with the fine pattern on the wall, 57 with the coarse one). Maximal number of flights per fly and pattern was 10 recording time per flight was limited to 16 s by the memory of the cameras. 1536 s of flight were analyzed in total (768 s with either fine or coarse pattern). In the analysis yaw rotations were defined to be saccadic turns if they exceeded a rotational velocity of 500°/s. A minimum distance criterion of 50 ms between saccade peaks was used to reject false detections. For each flight we calculated the average saccade frequency and average saccade peak velocity. We analyzed the spatial distribution of flight locations by computing the distance to the arena center for each recorded time step as well as for each detected saccade. Statistic tests were performed using the Matlab Statistics Toolbox (The Mathworks Inc., Natick, MA, USA).

## Results

### Model simulations

The CyberFly saccade generation algorithm proposed in Lindemann et al. (2008) generates saccades based on the signals of a pair of simulated LPTCs responding to front-to-back horizontal image movement in the equatorial region of the eyes, matching the properties of HSE neurons in the fly lobula plate. The timing of saccade generation was determined by applying a threshold operation to the signals. Saccade direction and amplitude depend on the relative difference of the two input signals. In the following we term this parameter the response contrast.

**Figure 1:**
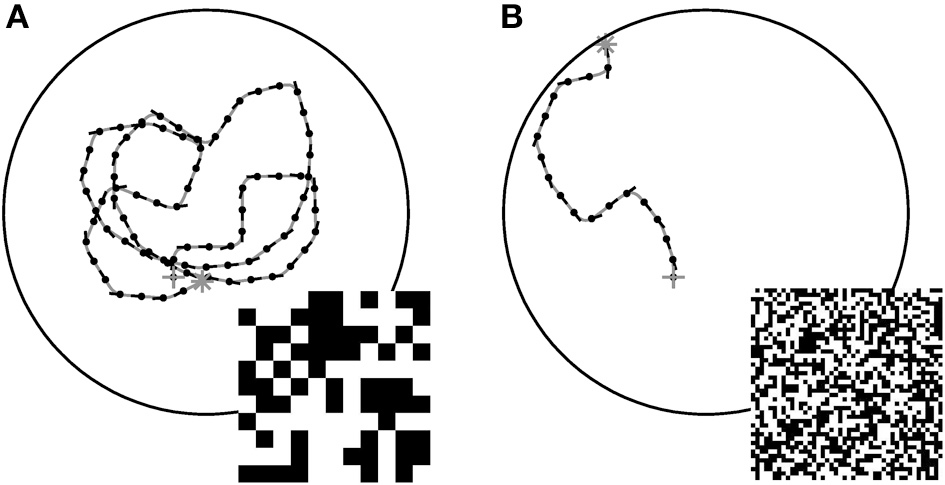
**Examples of closed loop trajectories.** Trajectories generated by the CyberFly algorithm in a virtual flight arena covered with textures of different texel size **(A)** coarse pattern, **(B)** fine pattern (see insets). The starting conditions were the same for the two flights. The change of texture density is sufficient to cause a severe drop in wall avoidance performance. Data from Lindemann et al. (2012).

The CyberFly was developed and parameterized using a random checkerboard texture composed of black and white squares with 10° edge length when viewed from the center of a cylindrical arena. In such an environment the system successfully avoids the walls confining the simulation space. However, replacing the texture by a similar texture composed of smaller texture elements, the CyberFly is unable to avoid the wall, but rather turns toward it (cf. Figure 1). Attempts to regain the performance with parameterization changes were not successful.

**Figure 2:**
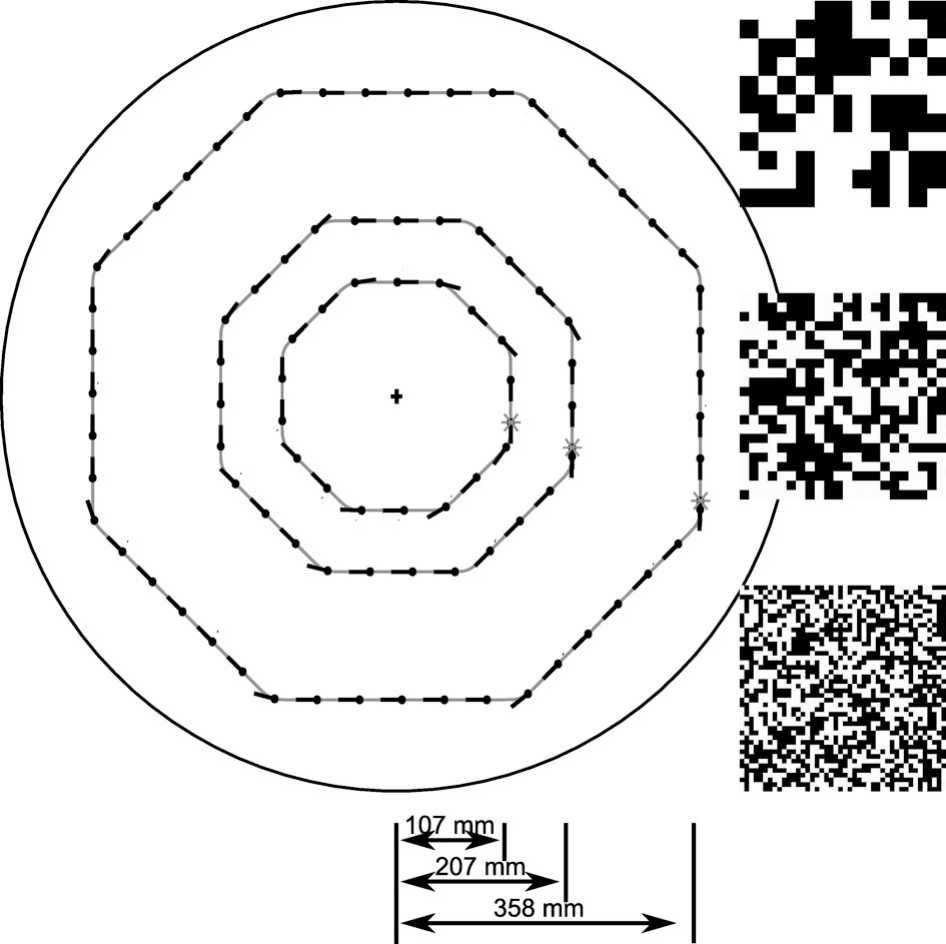
**Sketch of open-loop trajectories.** Trajectories used to systematically investigate the texture dependence of the sensory module of the CyberFly. The intersaccadic segments pair-wise differ by a factor of two in duration. The outmost trajectory was used in electrophysiology. Random checker-board patches represent the textures used for model analysis: Large, medium, and small texture elements pair-wise differ by a factor of two in edge length.

To systematically investigate the sensory responses leading to this unexpected behavior we created an open-loop stimulus that matches the characteristic saccadic flight structure generated by the CyberFly algorithm. Starting approximately parallel to the arena wall, the test trajectory is an octagonal flight path combined from segments of straight purely translatory flight alternating with saccadic turns of 45° amplitude. The spatio-temporal structure of all translational inter-saccadic segments is identical while the system is confronted with random changes in local structure of the texture segment in the receptive field of the simulated tangential cells. In this way we can analyze texture-dependent differences of the model cell responses between the segments at two scales: on the one hand, we can analyze how the responses change for the texture variants generated from differently sized squares. On the other hand, we can analyze how the responses depend on local random differences in a given texture by comparing the response across intersaccadic elements in one arena. We analyzed the open-loop situation with the two textures also tested in closed-loop and with a third texture composed of intermediate sized squares. For deeper analysis of the sensory module we further varied the length of the intersaccadic segment and the forward velocity (see Figure 2).

**Figure 3:**
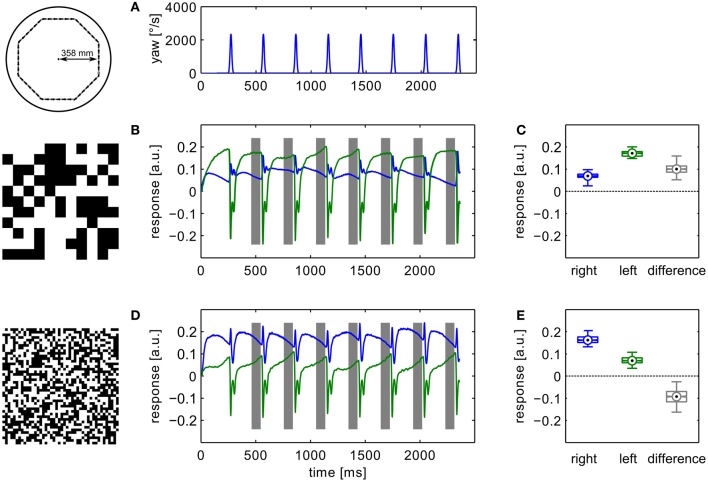
**Model responses.** Responses to the sensory open-loop stimuli generated on the flight trajectories shown in Figure 1 obtained in flight arenas covered with two different textures. Top panel **(A)** shows the yaw velocity versus time; it is characterized by regular saccadic turns interrupted by straight flight segments. Responses of the sensory module of CyberFly to open-loop stimulus shown in **(B,C)**, Figure 2. Response of the right input element shown in blue, response of the left element in green. In the boxplots **(D,E)**, the distribution of all right and left intersaccadic responses is shown in combination with the difference value used by CyberFly for saccade amplitude selection. The boxplots show median (black dot), quartiles (box), and the range of the data (whiskers). Non-overlapping notches indicate significant difference between two medians at a 5% significance level.

During translational movement between saccadic turns (see Figure 3A) the retinal velocities are inversely proportional to the wall distance. On our test trajectory the wall on the right side of the agent, being much closer than the opposite arena wall on the left, generates much higher retinal velocities. Based on the geometrical situation the retinal velocity can be computed for a given position in the trajectory, forward velocity, and retinal location (Koenderink and van Doorn, 1987). We calculated the retinal velocities for the central position in the intersaccadic segment on the outermost trajectory, 1 m/s forward velocity, and the retinal position of the maximum sensitivity of the simulated neuron (15° lateral on the eye equator). For the left eye facing the arena center this yields a retinal velocity of 36.8°/s. On the right eye the pattern moves with 68.0°/s. A motion sensor encoding velocity monotonically should thus respond with a larger model HSE signal on the right side of the CyberFly than on the left side. This is clearly not the case when tested with the coarse pattern (see Figures 3B,C). Here, the sensor viewing the wall responds with a smaller signal than its counterpart on the left side experiencing lower velocities. However, the change of textures causes an inversion of this situation. For the texture composed of small squares, the right model HSE cell, viewing the wall, responds more strongly than the left one (Figures 3D,E). This is consistent across the different repetitions of the translatory segments. In spite of small variations caused by the local structure of the random texture, the response contrast (i.e., the difference of the two responses normalized by their mean) changes in sign throughout the stimulus period. For the CyberFly algorithm this means that the saccade direction is inverted. Under geometrically identical conditions the algorithm generates leftward turns for one and rightward turns for the other texture.

**Figure 4:**
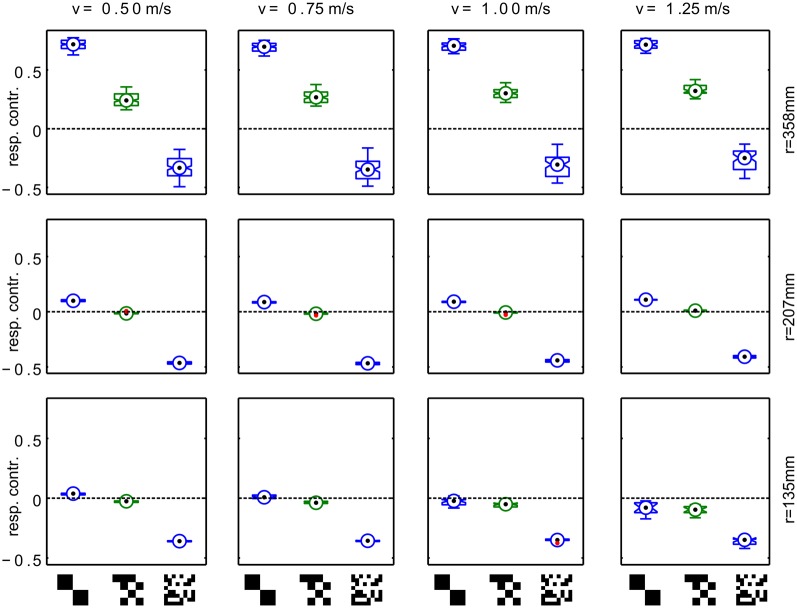
**Systematic variation of trajectory parameters.** Responses for trajectories with different radius(*r*) and translational velocity (*v*). Each panel shows the response contrast (difference of the HSE signals normalized by their sum) for textures composed of large, medium and small texels. The radius of the trajectories varies across rows, the forward velocity in the trajectory varies across columns. The boxplots show median (black dot), quartiles (box), and the range of the data (whiskers). Non-overlapping notches indicate significant difference between two medians at a 5% significance level.

Variation of the distance of the translational segments from the area center as well as changes in translation velocity within the plausible range (0.5–1.25 m/s) does not qualitatively alter this effect (Figure 4). A switch in sign of the response contrast can be observed for most tested parameter combinations with exception of the trajectory close to the arena center at high forward velocities. Here, we find consistently the response order as expected from the retinal velocities. However, even for high forward velocities the sign inversion of the response difference is obvious for trajectories closer to the wall. The response contrast for the texture composed of medium-sized squares shows intermediate values which means that the effect is continuously correlated with texture element size.

**Figure 5:**
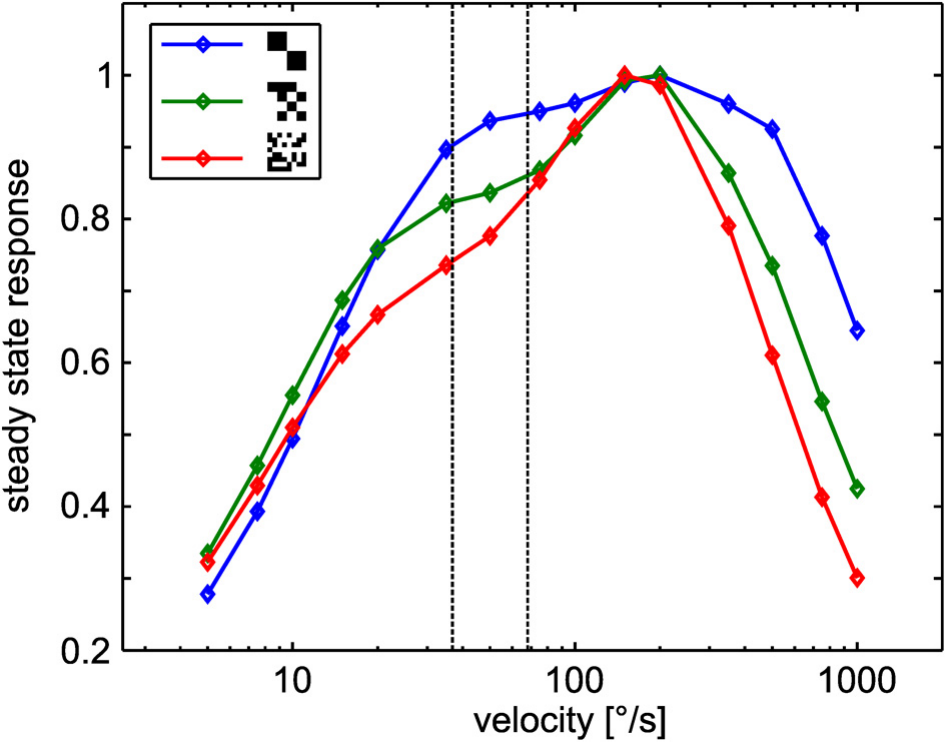
**Steady-state tuning of the sensory module.** EMD responses to constant angular velocity stimulation for the different textures. Data was normalized to maximum response obtained with each texture. The dotted lines indicate the retinal velocity range in the center of the receptive fields of the simulated neurons in the middle of the intersaccadic segment of the outmost trajectory (see text for details).

To check whether sign-reversal of the response contrast between the left and right model HSE cells can be explained by the general angular velocity tuning of the underlying correlation-type motion detectors, we tested the steady-state angular velocity tuning by constant velocity rotation in the center of the cylindrical arena with all three different textures. The resulting tuning curves show the typical bell-shaped tuning of the motion detector. Regardless of the texture variant, the responses peak at angular velocities of ~150°/s (Figure 5). Although the width of the tuning curves differs slightly, the gross shape is similar for all textures tested. This result is compatible with earlier reports (Dror et al., 2001; Straw et al., 2008) because, in spite of the different sizes of the basic elements, the textures share a broad spatial spectrum and have the same total contrast. Note that the steady-state tuning is not easy to interpret with respect to the CyberFly. First, the response of the motion detectors is sensitive to transient changes in stimulus angular velocity (Egelhaaf and Reichardt, 1987; Egelhaaf and Borst, 1989). Second, the retinal velocity varies substantially across the receptive field of the simulated neurons. Third, in the cylindrical environment, the retinal velocity distribution within the receptive field depends on the position in the inter-saccadic segment in a non-trivial way.

### Electrophysiological experiments

**Figure 6:**
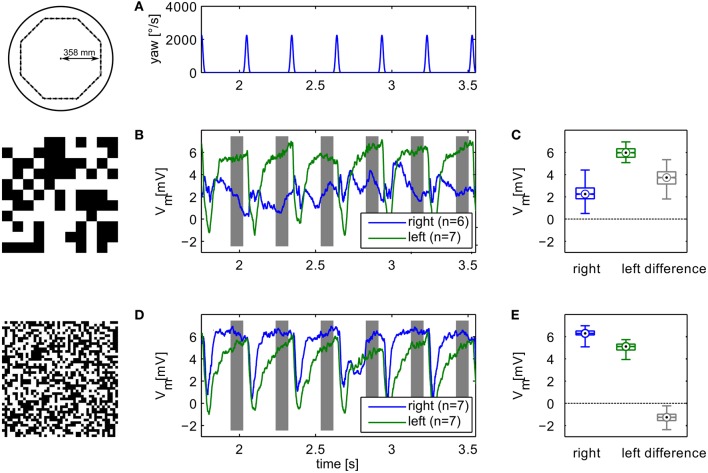
**Example of electrophysiological recordings.** Left column **(B,D)** shows membrane potential changes relative to the resting potential of the cell obtained with the different textures. Top panel shows the yaw velocity timecourse **(A)**. Right column **(C,E)** shows boxplots of the responses in the analysis windows indicated in grey in the time-courses. The boxplots show median (black dot), quartiles (box), and the range of the data (whiskers). Non-overlapping notches indicate significant difference between two medians at a 5% significance level. Compare to model responses shown in Figure 3.

In an electrophysiological control experiment we verified that the relevant behavior of the simulated HSE neurons concurs with the measured sensory properties of their counterparts in the flies' brain. We replayed image sequences rendered from an octagonal open-loop trajectory using the two extreme textures used in the simulation experiments to a blowfly and recorded the responses of the left and right HSE neurons. The resulting signals (Figure 6) show the same qualitative properties as we observed in simulation. The sign of the response contrast changes between textures. The figure shows the average responses for the recording yielding the most stimulus repetitions. The result was qualitatively confirmed in recordings from eight different animals.

The neuronal responses show strong variation of the signals also between intersaccadic segments. This might partly be explained by neural noise which is only insufficiently eliminated by averaging repeated recordings. However, some of the deviations can be observed consistently in trial-to-trial comparison. Since the retinal velocities are identical for each inter-saccadic segment, these response modulations thus reflect the local texture of the pattern covering the arena wall.

### Behavioral experiments

**Figure 7:**
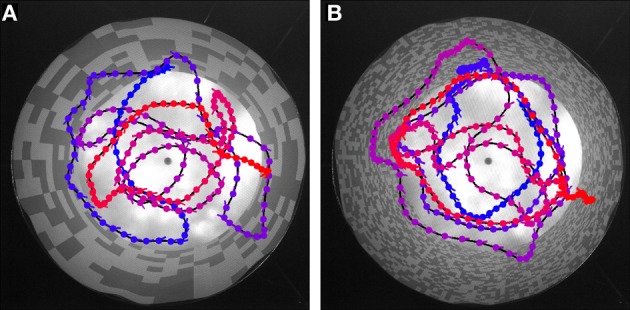
**Trajectory examples for behavior in the different textures.** The cylindrical arena is shown in top-view. **(A)** Coarse texture, **(B)** fine texture. Color in the trajectories indicates time: Blue indicates the start, red the final position. Blobs indicate fly position, line markers at each blob position indicate body orientation (pointing toward abdomen).

Flies are able to navigate without colliding with obstacles in very different spatial situations ranging from natural outdoor-environments to very artificial situations in offices or laboratory rooms. From this point of view, it would be surprising if flies have systematic problems in a cylindrical environment papered with any of the above described textures. Nevertheless, we did free flight experiments in an environment closely matched to the 3D those employed in the model simulations and the electrophysiological experiments. We again tested the two extreme textures and recorded the free flight behavior of six animals. As expected, the flies were able to safely avoid the walls for most of the time for both patterns. Figure 7 shows examples of flight trajectories (top-view). Note that due to the perspective distortion the distance of the fly from the wall cannot be estimated based on this view only. All flies showed the saccadic flight and gaze strategy characteristic of blowflies (Schilstra and van Hateren, 1999; van Hateren and Schilstra, 1999).

**Figure 8:**
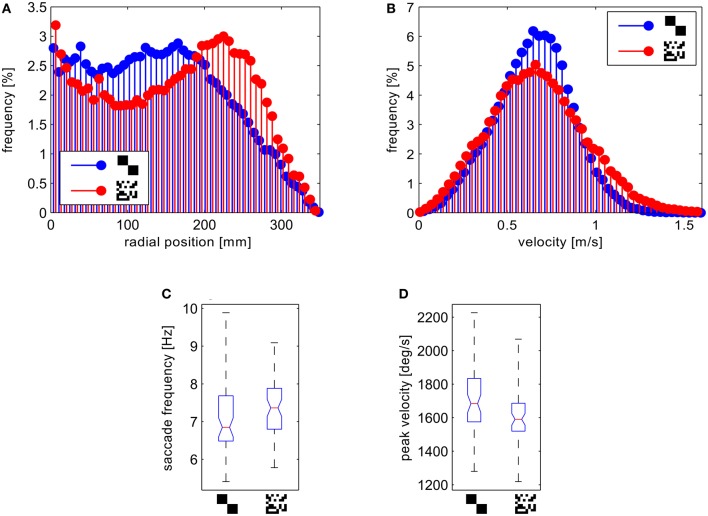
**Analysis of the behavioral data. (A)** Histograms of the distance of the fly position from the center normalized to the circumfence of each circular bin. **(B)** Histograms of the forward velocities. **(C)** Boxplots of the saccade frequency and **(D)** boxplots of mean saccade peak yaw velocities per flight. The boxplots show median (black dot), quartiles (box), and the range of the data (whiskers).

We analyzed and compared different aspects of the flight trajectories. Most of the flight parameters did not differ to a behaviorally relevant degree for the two wall textures. On average, the flies show very similar translational velocities (Figure 8B), as well as frequencies and amplitudes of saccadic turns (Figures 8C,D). Only the distributions of the distance to the wall differed significantly (two-sample Kolmogorov–Smirnov test, α = 0.01). The test for the distributions of the distance to the wall for the saccade locations leads to the same result.

For the fine texture flies flew on trajectories showing, on average, a slight preference for a certain distance from the arena wall. To test this statistically, we obtained the number of saccade locations in a central circular section and an outer ring of the arena for each flight, binning the distance to the arena center in two equal classes. We computed the relative proportion of saccade locations in the outer segment by calculating the ratio of these numbers. The distribution of these proportional values differed significantly between the two textures (two-sample Kolmogorov–Smirnov test, α = 0.01). In the arena covered with the fine texture we observed a higher proportion of saccades in the outer segment. Accordingly the histogram of the probability distribution of distances from the wall shows a maximum at ~15 cm from the wall for the fine texture. In contrast, the flies showed a more random probability of distances when confronted with the coarse texture (Figure 8A).

Although flies are generally viewed as aerobatic fliers, we observed several contacts with the arena wall without prior deceleration or leg extension. As a co-effect of the more random flight behavior in the arena papered with the coarse pattern, we observed these collisions with the wall more frequently than in the setup papered with small texture elements.

## Discussion

Many aspects of the behavioral and neural responses of flies to motion stimuli can be described by a well-known algorithmic model, the correlation motion detector (Hassenstein and Reichardt, 1956; Reichardt, 1961). In various variants, this model can account for the results of behavioral experiments as well as for responses of large motion-sensitive neurons in the third optic lobe of the fly (reviews: Egelhaaf, 2006; Borst and Haag, 2007; Borst et al., 2010; Egelhaaf et al., 2012). The model and its biological counterpart share the important property that the motion-dependent response not only reflects the angular velocity of a stimulus but also depends on stimulus parameters unrelated to visual movement, such as the contrast and spatial composition of the pattern (Egelhaaf et al., 1989; Meyer et al., 2011; O'Carroll et al., 2011). Under dynamic stimulation the transient properties of correlation-type motion detectors (Egelhaaf and Reichardt, 1987; Egelhaaf and Borst, 1989) further complicate the interpretation of responses as obtained under dynamical stimulus conditions characteristic of free flight (Kern et al., 2005).

A simulation model of the HSE neuron of the fly was used as the sensory input stage of a closed-loop model of collision avoidance behavior (CyberFly; Lindemann et al., 2008). The CyberFly was designed to avoid the wall of a flight arena based on the relative difference between responses of the two model HSE neurons detecting front-to-back motion on the left and right side of the animal, respectively. The model was tuned to show good performance in collision avoidance in a cylindrical setup papered with a random checkerboard texture. However, reducing the size of the texture elements led to an unexpectedly strong reduction in closed-loop system performance and to very frequent crashes with the arena wall.

Here we show by systematic analysis of model HSE neurons and their biological counterparts with open-loop stimulation that this loss of performance can be explained by a texture-dependent inversion of the sign of the difference of the responses of HSE-cells on the left and the right side. Because the sign of the response difference determines the direction of saccadic turns in our model, this causes a switch from wall avoidance to wall attraction with a given set of parameters.

The most obvious difference between the HSE model as used in the simulations and its neuronal counterpart is the contrast dependence of the response. The neuron is known to show contrast saturation and adaptation. This property was addressed by elaborated models of the motion detection pathway (Brinkworth and O'Carroll, 2009). However, the model used here shows the quadratic dependence of the motion detector responses on contrast (Egelhaaf et al., 1989). Using a contrast normalizing elaboration of the motion detector (Babies et al., 2011) we checked that the sign-reversal effect is not only a contrast effect (data not shown). Moreover, the existence of texture-dependent response fluctuations of model HSE neurons persist, in accordance with the properties of biological HSE neurons (O'Carroll et al., 2011), even in model versions in which the non-linear contrast transfer in the peripheral visual system of the fly was taken into account (Meyer et al., 2011).

In an electrophysiological control experiment we further showed that the sign inversion observed in the model is not an artifact of the model but can also be observed in the response of real neurons stimulated with the same stimuli. Recent findings suggest that the responses of lobula plate tangential cells depend on the behavioral state of the animal (for review see Maimon, 2011). We do not know the behavioral state of the flies in our electrophysiological experiment. However, the response changes caused by different activity states are too subtle to explain the qualitative change observed here.

Our electrophysiological results indicate that the signals in the motion vision pathway depend on textural properties of the wall of the flight arena in such a strong way that comparison of the amplitude of these signals from the two visual hemispheres, as proposed for the CyberFly, can lead for different textures to qualitatively varying results. Depending on the texture, the direction of a saccade generated by the CyberFly algorithm is inverted. This is unexpected, because the geometric situation leads to identical optic flow fields and because changing the size of the squares composing the random checkerboards does not result in a very pronounced change in the spatial frequency composition of the texture. Earlier studies suggest that the responses therefore should not be qualitatively different (Dror et al., 2001).

As expected from many behavioral experiments in flight arenas covered with a wide range of textures (Kern et al., 2000, 2012; Tammero and Dickinson, 2002a) flies are well able to deal with all scales of random checker board textures without colliding with the arena walls. This finding is corroborated by the behavioral results of our study. In particular, they can deal with the whole range of textures used in our modeling and electrophysiological open-loop tests. Nevertheless, some texture dependence in the flight details is obvious. Flies tended to keep a more consistent distance to the wall for the texture composed of small squares and collided with the wall more often when it was papered with larger squares. Earlier studies on *Drosophila* free flight behavior also document dependencies on wall texture, although for much more radical texture changes (Tammero and Dickinson, 2002b; Frye and Dickinson, 2007). We therefore infer that fly motion vision is strongly texture-dependent and that some of this dependence can be observed on the behavioral level. This suggests that the visual motion pathway of the fly is not optimized to reflect motion information in a texture-independent way.

Texture dependence is an inherent property of the motion detection mechanism of insects and can be accounted for by correlation-type movement detectors. Spatial integration of many local motion detectors reduces the texture dependence of the response. Full elimination of texture-dependent response fluctuations requires integration across the full visual field (Meyer et al., 2011). However, any saccade triggering mechanism based on visual motion information can be expected to receive input from cells integrating motion information from only parts of the visual field, because it somehow needs to extract asymmetries in the optic flow in front of the two eyes. In *Drosophila*, expansion detectors in the fronto-lateral visual field have been suggested on the basis of behavioral experiments to control saccadic turns in the context of collision avoidance behavior (Tammero and Dickinson, 2002a,b). A robotic implementation of a model based on these experiments was shown to be successful in avoiding the walls of a cylindrical environment with a given texture (Reiser and Dickinson, 2003). Since the expansion detector implemented in that system spatially integrate responses of correlation-type movement detectors, their responses can be assumed to be affected, in a similar way as in our CyberFly model, by the textural properties of the environment. Furthermore, *Drosophila* and the blowfly *Lucilia* that we used in our experiments show different flight styles. In contrast to *Drosophila* which may hover on the spot and shows pronounced sideways movements, blowflies reveal large sideways components only rarely; rather the forward component dominates almost always between saccadic turns (Schilstra and van Hateren, 1999; van Hateren and Schilstra, 1999; Braun et al., 2010; Kern et al., 2012). Thus, lateral expansion detectors are an unlikely mechanism for saccadic control in blowflies in the context of obstacle avoidance. Nonetheless, irrespective of the exact location of such expansion detectors, if these detectors are based on correlation-type movement detectors, their responses can be expected to depend on texture like HSE cells, a prediction that has not been tested so far.

Image expansion plays a role also in another behavioral context, i.e., landing behavior: tethered flying flies extend their legs as if they would prepare for landing in response to image expansion in the frontal visual field. This landing response was found to depend also on the textural properties of the expanding stimulus and concluded to be based on correlation-type movement detectors (Borst and Bahde, 1987; Borst, 1990). However, different computational principles may be involved in yet another behavioral context, i.e., the escape response to approaching objects. By a combination of physiological and optogenetic techniques visual neurons could be identified that detect approaching objects, and whose activation could be shown to play a role in mediating escape responses of sitting animals to looming stimuli (de Vries and Clandinin, 2012). These neurons were concluded to have similar properties to the long-established LGMD/DCMD system of locust (Fraser Rowell et al., 1977; Rind, 1987; Peron et al., 2009). The properties of these neurons are likely to differ substantially from those of the LPTCs, such as HSE, of flies and have been interpreted by other computational models. Still it is not yet clear to what extent the responses of these neurons are affected by the textural properties of looming stimuli, on the one hand, and whether they are only involved in mediating escape responses to approaching objects, or also play a role in avoiding collisions of flying animals with spatially extended obstacles, on the other hand.

If saccadic turn decisions mediating collision avoidance with spatially extended obstacles may be based on optic flow information extracted by correlation-type movement detectors and further processed by LPTCs, such as the HS-cells analyzed here, the input to the sensory-motor interface is strongly pattern dependent. Since behavior depends only little on pattern properties flies then should have evolved a sensory-motor coupling which compensates for the textural dependence of the motion signals either by combining several motion-sensitive information channels like the signals of an ensemble of LPTCs or by including complementary visual information not represented in the motion pathway. Partial compensation of texture dependence of the motion signals can be accomplished, for example, by a mechanism proposed recently for robotic vision (Wu et al., 2012). In analogy to this mechanism, an independent pathway could provide texture information, which might then be combined with the motion signals provided by LPTCs to generate unambiguous angular velocity information. However, so far, there is no evidence that such a mechanism exists in the fly brain. Furthermore, it is possible that saccades in the context of collision avoidance are controlled by a visual pathway, parallel to the known visual motion pathway experimentally analyzed and modeled here, that extracts information in a texture-independent way from the retinal image. There is evidence for a second motion processing pathway from a neurogenetic and behavioral study on orientation responses of walking *Drosophilae* (Katsov and Clandinin, 2008). Still, it is currently not clear whether its responses are independent of the textural properties of the environment and whether this pathway plays a role in saccade control of flying flies.

We conclude that the problem of how saccade generation in the context of collision avoidance is controlled largely independent of the texture of obstacles in the environment can only be resolved to some extent on the basis of current experimental evidence, and further analyses are required.

### Conflict of interest statement

The authors declare that the research was conducted in the absence of any commercial or financial relationships that could be construed as a potential conflict of interest.
